# Two new non-chlorophyll *f*-producing species in the *Kovacikia* genus (Leptolyngbyaceae, Leptolyngbyales) from the Poyang Lake Basin, China

**DOI:** 10.3389/fmicb.2025.1578689

**Published:** 2025-09-22

**Authors:** Li-Qin Shen, Guofei Dai, Ying Le, Sheng Lai, Xinyuan Liu, Renhui Li, Jutao Liu

**Affiliations:** ^1^Jiangxi Provincial Technology Innovation Center for Ecological Water Engineering in Poyang Lake Basin, Jiangxi Key Laboratory of Flood and Drought Disaster Defense, Jiangxi Academy of Water Science and Engineering, Nanchang, China; ^2^College of Life and Environmental Science, Wenzhou University, Wenzhou, China

**Keywords:** cyanobacteria, *Leptolyngbya*-like, *Kovacikia*, new species, polyphasic approach, chlorophyll *f*

## Abstract

For the filamentous cyanobacteria, the order Leptolyngbyales is the larger and obviously polyphyletic group, although its members are morphologically similar. In this study, three *Leptolyngbya*-like strains were isolated from the soil of three different habitats in the Poyang Lake Basin, China—dark microhabitats similar to those found in far-red light (FRL) habitats. The three strains were phylogenetically identified as *Kovacikia diezihuensis* sp. nov. and *Kovacikia jiangxiensis* sp. nov. (Leptolyngbyaceae, Leptolyngbyales) using a polyphasic approach, respectively. The phylogenetic tree showed that they clustered within the *Kovacikia* genus (species type: *Kovacikia muscicola*) with five other *Kovacikia* species. The *16S rRNA* gene sequences of *K. diezihuensis* and *K. jiangxiensis* shared 97.2% similarity with each other (data not shown) and 94.9–96.8% similarity with five other *Kovacikia* species. Furthermore, the secondary structures of the internal transcribed spacer (ITS) regions and ITS sequences exhibited uniqueness. The two species were similar to the other five *Kovacikia* species in morphology, but *K. diezihuensis* was bright blue-green, and *K. jiangxiensis* was gray-green to blue-green in color, and its length was usually greater than its width in cells under white light, respectively. Pigment analysis showed that the two strains did not produce phycoerythrin. FRL adaptation experiments further showed that they could neither grow nor produce chlorophyll (Chl) *f* under FRL. In summary, *K. diezihuensis* and *K. jiangxiensis* were new non-Chl *f*-producing species in the *Kovacikia* genus. This is the first report of both Chl *f*-producing and non-Chl *f*-producing species in the same genus within the Leptolyngbyales, shedding light on the diversity and the evolutionary divergence of Chl *f*-producing cyanobacteria.

## 1 Introduction

Cyanobacteria, as a highly diverse group, have long faced challenges in phylogenetic studies at the genus and subgenus levels (Hoffmann et al., 2005). In the past, classifying cyanobacteria solely based on their morphological characteristics led to a serious underestimation of cyanobacterial diversity (Taton et al., 2003; Komárek, 2006; Engene et al., 2011). Among them, filamentous cyanobacteria are particularly difficult to classify due to their subtle morphological differences and diverse species. The introduction of molecular genetics methods and electron microscopy has fundamentally driven the transformation of cyanobacteria classification (Komárek et al., 2014; Komárek, 2016). For example, Komárek et al. (2014) revised the classification of cyanobacteria, dividing Oscillatoriales into Oscillatoriales and Synechococcales, based on the preliminary results of phylogenetic analyses and the ultrastructural patterns of thylakoids. The filamentous cyanobacteria, *Leptolyngbya* (Anagnostidis and Komárek, 1988), are therefore classified into the new family Leptolyngbyaceae, Synechococcales. In recent years, with the increasing number of reference strains of currently identified cyanobacteria and well-described genome sequences, Strunecký et al. (2023) reconstructed a robust phylogenomic tree to classify the entire cyanobacteria phylum, including 10 new orders and 15 new families. Therefore, many *Leptolyngbya*-like cyanobacteria no longer belong to the Synechococcales but to Leptolyngbyales (including Leptolyngbyaceae, Trichocoleusaceae, and Neosynechococaceae), Oculatellales, and Nodosilineales.

*Leptolyngbya* is a larger and obviously polyphyletic group, but it is almost impossible to separate it based on morphological characteristics (Komárek, 2016). A polyphasic approach combining ecological, molecular, and morphological data has been more recently applied, providing more evidence for the genera and species levels classification (Iii et al., 2011; Vaz et al., 2015; Engene et al., 2018). The traditional definition of *Leptolyngbya* actually includes several distinct and not closely related phylogenetic clusters, some of which have been formally identified as new genera separated from *Leptolyngbya* by polyphasic approach, such as *Phormidesmis* (Turicchia et al., 2009; Raabová et al., 2019), *Tapinothrix* (Bohunická et al., 2011), *Alkalinema* (Vaz et al., 2015), *Pantanalinema* (Vaz et al., 2015), *Scytolyngbya* (Song et al., 2015), *Limnolyngbya* (Li and Li, 2016), *Kovacikia* (Miscoe et al., 2016; Shen et al., 2022; Kaštovský et al., 2023), *Stenomitos* (Miscoe et al., 2016; Shalygin et al., 2020), *Chamaethrix* (Dvořák et al., 2017), *Onodrimia* (Jahodářová et al., 2017), *Chroakolemma* (Becerra-Absalón et al., 2018), *Myxacorys* (Pietrasiak et al., 2019; Soares et al., 2019), *Leptodesmis* (Raabová et al., 2019; Strunecky et al., 2020; Tang et al., 2022; Luz et al., 2023; Kaštovský et al., 2023; Shen et al., 2024), *Apatinema* (Davydov and Vilnet, 2022), *Romeriopsis* (Hentschke et al., 2022), *Khargia* (Rasouli-Dogaheh et al., 2023), *Pseudoleptolyngbya* (Hentschke et al., 2024), and *Leptolyngbyopsis* (Hentschke et al., 2024). It is worth noting that there are relatively few chlorophyll (Chl) *f*-producing studies in the aforementioned cyanobacteria.

Chl *f* was the newest member of the Chl family, and was a far-red light (FRL) induced Chl (Chen et al., 2010, 2012). Cyanobacteria restructured their photosynthetic apparatus and produced Chl *f* through a complex and extensive light adaptation reaction called FRL photoadaptation (FaRLiP), greatly improving their photosynthetic performance under FRL and playing an important ecological role as primary producers under FRL environment (Gan et al., 2014; 2015; Li et al., 2016; Kato et al., 2020; Gisriel et al., 2020, 2022a, 2022b). At present, it is known that Chl *f* only exists in some cyanobacteria, and these cyanobacteria that can produce Chl *f* including *Halomicronema* (Li et al., 2014), *Aphanocapsa* (Akutsu et al., 2011; Miyashita et al., 2014; Zhang et al., 2019), *Chlorogloeopsis* (Airs et al., 2014; Gan et al., 2014), *Leptolyngbya* (Gan et al., 2014; Ohkubo and Miyashita, 2017; Zhang et al., 2019), *Calothrix* (Gan et al., 2014), *Fischerella* (Gan et al., 2015), *Synechococcus* (Gan et al., 2015), *Chroococcidiopsis* (Gan et al., 2015; Zhang et al., 2019), *Altericista* (Averina et al., 2021), “*Leptothermofonsia*” (Tang et al., 2022), *Kovacikia* (Shen et al., 2022), *Elainella* (Shen et al., 2023), *Pegethrix* (Shen et al., 2023), and *Leptodesmis* (Shen et al., 2024). This indicates that Chl *f*-producing cyanobacteria possess significant diversity and warrant further taxonomic research. Recently, a polyphasic approach has also been gradually applied to the classification of Chl *f*-producing cyanobacteria, and some new species have been successfully identified, such as *Altericista* var*iichlora* (Averina et al., 2021), *Kovacikia minuta* (Shen et al., 2022), *Pegethrix sichuanica*, and *Elainella chongqingensis* (Shen et al., 2023), and four new species of the *Leptodesmis* genus (Shen et al., 2024).

In this study, three *Leptolyngbya*-like cyanobacterial strains were isolated from the Poyang Lake Basin, China. Their preliminary molecular data showed they are genetically diverse, forming a new clade in *Kovacikia* that deserves further investigation regarding their taxonomic status. Therefore, a polyphasic approach was adopted to classify the three strains based on comprehensive morphological characters, *16S rRNA* gene sequences phylogeny and sequence similarity, internal transcribed space (ITS) secondary structure, and ecological characters. Finally, these three strains were classified as *Kovacikia diezihuensis* sp. nov. (reference strain, ACCP0342) and *Kovacikia jiangxiensis* sp. nov. (reference strain, ACCP0444) by a polyphasic approach. Since the three strains were isolated under white light (WL), it is speculated that they might lack the ability to produce Chl *f* under FRL. Therefore, FRL adaptation experiments were designed, revealing that *K*. *diezihuensis* and *K. jiangxiensis* could neither produce Chl *f* nor grow under the induction of FRL. Up to now, five species have been reported in the *Kovacikia* genus, including *Kovacikia muscicola* (type species) (Miscoe et al., 2016), *K. minuta* (Shen et al., 2022), *Kovacikia anagnostidisii* (Kaštovský et al., 2023), *Kovacikia brockii* (Kaštovský et al., 2023), and *Kovacikia atmophytica* (Luz et al., 2023). Following the discovery of Chl *f*-producing species *K. minuta* in 2022, this study found that *K. diezihuensis* and *K. jiangxiensis* were the only two new species in the *Kovacikia* genus that do not produce Chl *f*. This discovery indicates that Chl *f*-producing and non-Chl *f*-producing cyanobacteria can belong to different species at the same genus level in the Lepolyngbylales. This provides significant clues for further exploring the evolutionary divergence of Chl *f*-producing cyanobacteria and their adaptability to the environment.

## 2 Materials and methods

### 2.1 Strain isolation, purification, and maintenance

**Figure 1:**
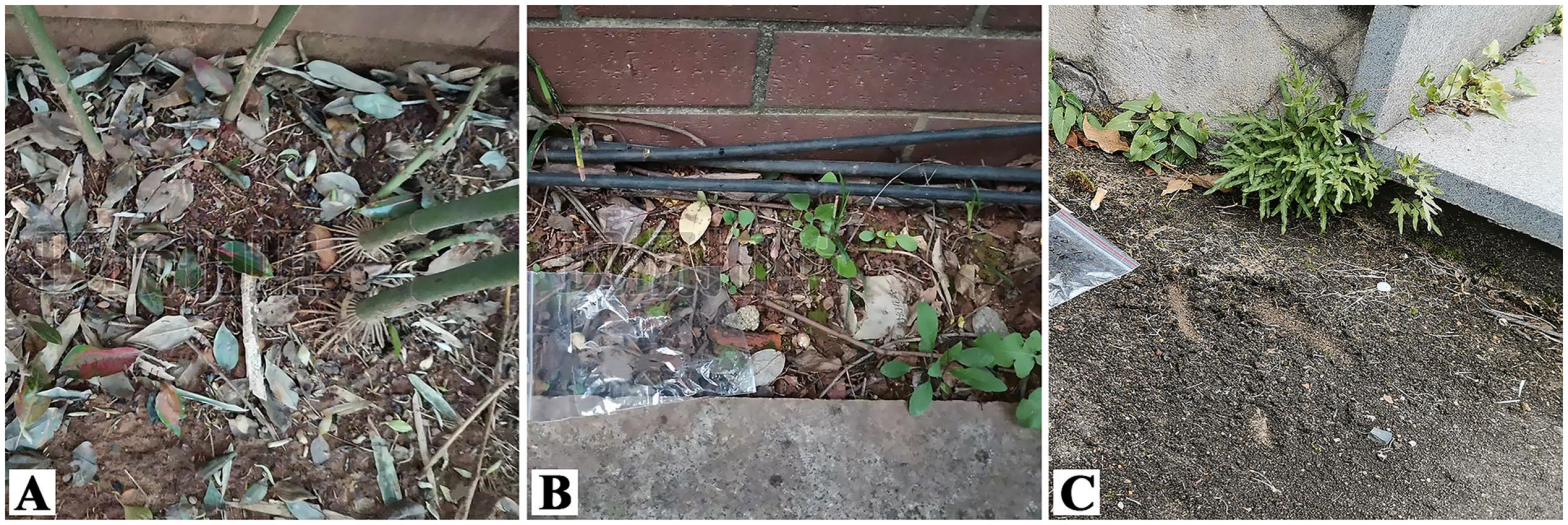
Strain habitats. **(A)** (28°41′12.13″N, 115°50′06.57″E), the soil under the bamboo forest in the corner at Diezihu avenue, Nanchang City, Jiangxi Province; **(B)** (28°41′12.32″N, 115°50′05.56″E), moss-like soil in the corner of the wall at Diezihu Avenue, Nanchang City, Jiangxi Province; **(C)** (28°41′0.786″N, 116°1′50.467″E), dried fluffy soil in the corner of Jiangxi Normal University, Nanchang City, Jiangxi Province.

The samples used in this study were isolated from the soil surface under a bamboo forest in the corner of a wall at Diezihu Avenue, Nanchang City, Jiangxi Province (28°41′12.13″N, 115°50′06.57″E), moss-like soil in the corner of a wall at Diezihu Avenue, Nanchang City, Jiangxi Province (28°41′12.32″N, 115°50′05.56″E), and dried fluffy soil in the corner of Jiangxi Normal University, Nanchang City, Jiangxi Province (28°41′0.786″N, 116°1′50.467″E) (Figure 1, Table 1). These were shadowy environments similar to an FRL habitat.

**Table 1:** Basic information on sample collection and pure algae strains.

Species name	Strains number	Collection sites and habitats	Longitudes and latitudes	Sampler and sampling time	Reference
*Kovacikia diezihuensis*	ACCP0342 (Reference strain)	Moss-like soil under the bamboo forest in the corner of the wall at Diezihu Avenue, Nanchang City, Jiangxi Province.	28°41′12.13″N, 115°50′06.57″E	Xing-Yuan Wu; 15 March 2021	This study
	ACCP0340	Moss-like soil in the corner of the wall at Diezihu Avenue, Nanchang City, Jiangxi Province.	28°41′12.32″N, 115°50′05.56″E	Xing-Yuan Wu; 15 March 2021	This study
*Kovacikia jiangxiensis*	ACCP0444 (Reference strain)	Dried moss-like soil in the corner of Jiangxi Normal University, Nanchang City, Jiangxi Province.	28°41′0.786″N, 116°1′50.467″E	Xing-Yuan Wu; 30 December 2020	This study
*Kovacikia muscicola*	HA7619-LM3	Growing in Waikapala’e Cave, associated with moss growing on the cave walls.	22°13′13″N, 159°34′46″W	July 2009, January 2011, May 2011, and January 2012	Miscoe et al. (2016)
*Kovacikia minuta*	CCNU0001	Underneath macrophytes in a shaded pond in the Wuhan Botanical Garden, Hubei Province, China.	30°32′43.30″N, 114°24′57.71″E	Zhong-Chun Zhang, Zheng-Ke Li, and Yan-Chao Yin; 27 September 2016	Shen et al. (2022)
*Kovacikia anagnostidisii*	YS86-RH1	Atmophytic, in massive ropy crust close to water, ca. 85 °C. Clearwater Springs (Yellowstone National Park, WY, USA).	44.7886475 N, 110.7392136 W.	18 September 2019 by Jeffrey R. Johansen, Jan Kaštovský, and Jan Mareš	Kaštovský et al. (2023)
*Kovacikia brockii*	YNP74-RH1	Atmophytic mats near the shore of the hot water. Firehole Lake (Yellowstone National Park, WY, USA).	44.5440433 N, 110.7865600 W.	18 September 2019 by Jeffrey R. Johansen, Jan Kaštovský, and Jan Mareš	Kaštovský et al. (2023)
*Kovacikia atmophytica*	BACA0619	Freshwater, atmophytic among other cyanobacteria and microalgae. At or near the top of a deep fumarole at Furnas do Enxofre, Terceira Island, Azores archipelago, Portugal.	38°43.733′N, 27°13.892′W.	4 March 2020 by Martin Souto, Pedro R. Raposeiro and Ana Balibrea.	Luz et al. (2023)

The appropriate samples were added to 100 mL sterile BG11 culture medium (Ichimura, 1979) in the 250 mL conical flask and incubated at 25 °C and 5–10 μmol photons m^−2^ s^−1^ under WL until the culture appeared. Then the cultures were homogenized to disperse the cells as evenly as possible and purified using the dilution spread plate method on a 0.8% solid BG11 plate. Finally, unialgal strains were successfully isolated and expanded in BG11 culture medium. The purity of the isolated strains was determined using an inverted microscope (Olympus, Tokyo, Japan). The pure strains were maintained in the 250 mL conical flask containing 120 mL BG11 medium under WL, respectively, at the Algae Culture Collection of Poyang Lake (ACCP), Jiangxi Key Laboratory of Flood and Drought Disaster Defense, Jiangxi Academy of Water Science and Engineering (JAWSE).

### 2.2 PCR amplification and phylogenetic analysis

Cyanobacterial genomic DNA was extracted using the modified version of the CTAB method (Neilan et al., 1995). The oligonucleotide primers of PA (5′-AGAGTTTGATCCTGGCTCAG-3′) and B23S (5′-CTTCGCCTCTGTGTGCCTAGGT-3′) were used to amplify the *16S rRNA* gene and the complete *16S–23S rRNA* internal transcribed spacer (ITS) region (Edwards et al., 1989; Lepère et al., 2000). The polymerase chain reaction (PCR) reaction system comprised 25 μL of PCR Mix 2 × Taq Plus Master Mix II (Dye Plus, P213-01/02/03, Vazyme, Nanjing, China), 2 μL of each forward and reverse primer, 2 μL of DNA, and sterile water to a final volume of 50 μL. The thermal cycling conditions were set to an initial denaturation step at 95 °C for 5 min; 35 cycles of DNA denaturation at 95 °C for 30s, primer annealing at 58 °C for 30 s, and strand extension at 72 °C for 2 min 30 s; and a final extension at 72 °C for 10 min. The PCR products were detected by electrophoresis and were then recovered. The recovered products were ligated with the pMD™ 18-T vector (Takara, Japan) and then transfected into competent *Escherichia coli* cells. Cloning experiments were performed according to the manufacturer’s protocol. Finally, positive single clones were sequenced by Sangon Biotech (Shanghai, China). The sequences were processed using BioEdit 7.2.5 (Hall, 1999) software and uploaded to NCBI GenBank, with accession numbers PQ881585, PQ881586, and PQ895252, respectively.

The *16S rRNA* gene sequences were aligned using BioEdit 7.2.5 (Hall, 1999). The best-fit models under the Akaike Information Criterion (AIC), estimated by ModelFinder (Kalyaanamoorthy et al., 2017), were adopted for both Bayesian inference (BI) and maximum likelihood (ML) analyses, respectively. The particular parameters of the substitution model for BI and ML were individually estimated using MrBayes version 3.2.6 (Ronquist et al., 2012) and IQ-TREE version 1.5.6 (Nguyen et al., 2015), respectively. For the BI analysis, two independent runs of four Markov chains were executed for 10 million generations with sampling every 100 generations. A total of 1,000 bootstrap replicates were conducted to evaluate the relative support of branches. Bootstrap analysis was performed with 1,000 replicates for the ML phylogenetic tree to estimate the degree of confidence for each branch node. Phylogenetic relationships of the *16S rRNA* genes were also analyzed by neighbor-joining (NJ) phylogenetic trees (Shen et al., 2023). *Gloeobacter violaceus* PCC 7421 was used as the outgroup. The three different methods yielded the same phylograms on the branches that gave the bootstrap values. FigTree version 1.5.6 was used to visualize the phylogenetic tree (Nguyen et al., 2015). Bootstrap values greater than 70% with BI/ML/NJ methods are shown in the Bayesian phylogenetic tree. The sequence similarity of the *16S rRNA* gene was calculated in MEGA.

### 2.3 Analysis of ITS secondary structure

The *16S–23S rRNA* sequences were aligned using BioEdit 7.2.5 (Hall, 1999) to determine similarity and dissimilarity percentages. The sequences of *16S–23S rRNA* ITS regions were also utilized for taxonomic resolution of the strains under investigation at the species level. The complete ITS sequences for these strains were aligned with the corresponding sequences, and different conserved and variable regions were identified in accordance with the method described by Iteman et al. (2000). The secondary structure of the ITS regions, such as D1–D1’, Box-B, and V3 helices, were predicted with the m-Fold web server (Zuker, 2003), and each fragment was folded individually. Default parameters were used in this study.

### 2.4 Morphological and ultrastructural examination

An appropriate amount of homogenate of algal filaments was added to the fresh liquid BG11 medium and grown for approximately two weeks at 25 °C and 5–10 μmol photons m^−2^ s^−1^ under WL. The cultures were examined using a Nikon Eclipse 80i light microscope with an external digital camera of Nikon DS-Ri1 (Tokyo, Japan). At least 100 measurements were obtained for cell length and width from at least 10 different algal filaments. Colony morphology was recorded using a Nikon D5600 digital camera. The subcellular ultrastructure was examined using transmission electron microscopy (TEM) (Hitachi HT-7700, Tokyo, Japan) according to a previously described method (Shen et al., 2022).

### 2.5 Pigment compositions of the new isolates

The absorption spectra of cultured algal filaments in good condition, as described above, including the cyanobacterial culture and lipid-soluble and water-soluble pigments, were analyzed in the range of 400–800 nm using an Integrating Sphere Ultraviolet (UV) Spectrophotometer (Specord 210 Plus; Analytik Jena, Jena, Germany). All operations were conducted under dim or dark conditions. Water-soluble pigments were extracted by phosphate buffer with appropriate modifications (Bennett and Bogorad, 1973; Shen et al., 2024). Lipid-soluble pigments were extracted by methanol with appropriate modifications (Zhang et al., 2019). The algae were centrifuged at 4 °C, 13,200*g* for 2 min, and the resulting supernatant was removed, and the algal pellet was retained. Fifty percent methanol was added, the samples were mixed, and the supernatant was removed after centrifugation. Pre-cooled (at −20 °C) 100% methanol was added, and the algae were extracted overnight at −20 °C. After centrifugation at 4 °C, 13,200*g* for 2 min, the supernatant was collected for absorption spectrum analysis of lipid-soluble pigments.

## 3 Results

### 3.1 Phylogenetic evaluation

**Figure 2:**
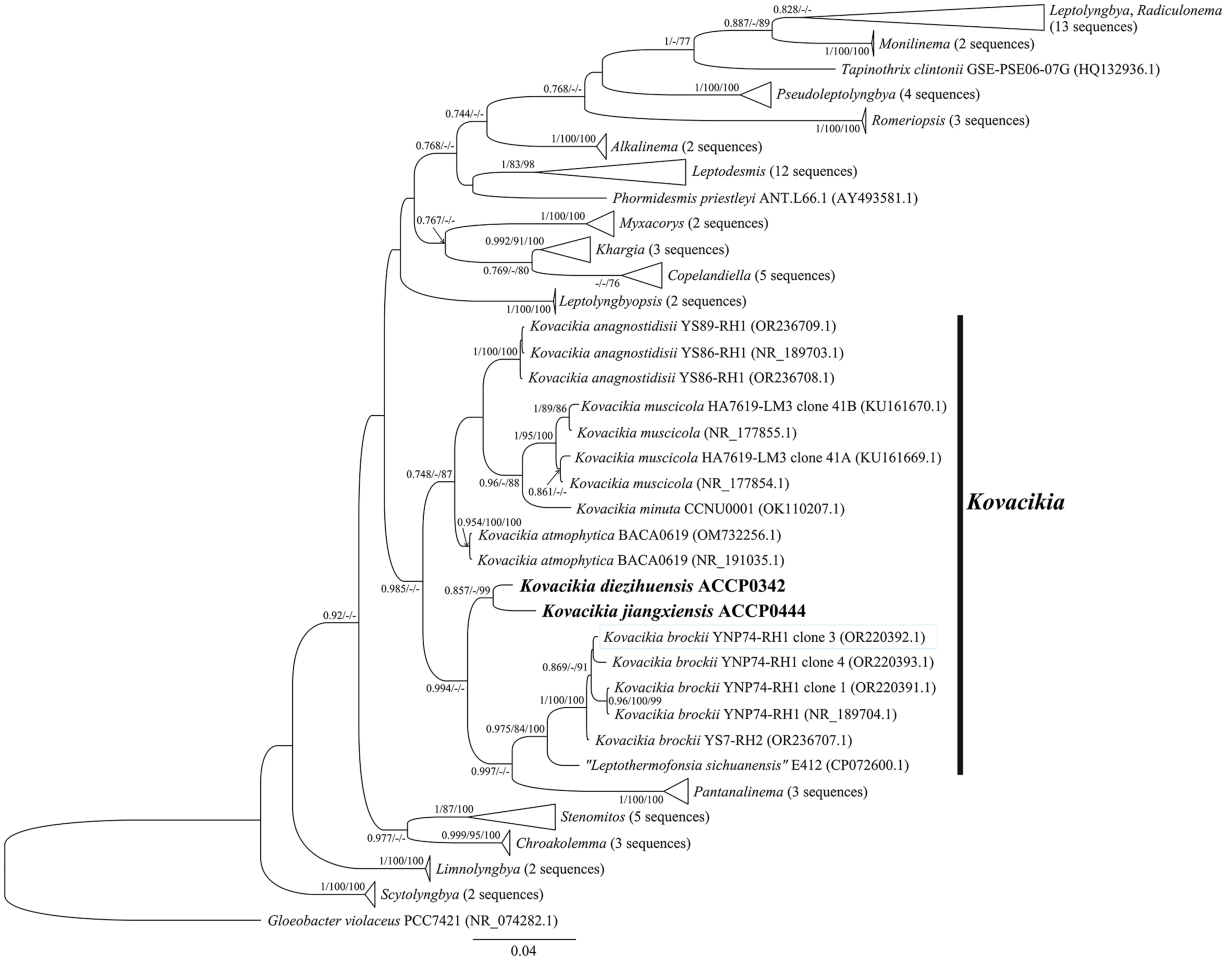
Bayesian phylogenetic tree based on the *16S rRNA* gene sequences showing the relationships of *Kovacikia diezihuensis* ACCP0342 and *Kovacikia jiangxiensis* ACCP0444 (bold part) with their similar taxa. The nodes marked by black dots were genus horizontal, and the black square nodes were horizontal nodes of the Leplyngbyaceae family. Bootstrap values greater than 70% were given in front of the corresponding nodes for BI/ML/NJ phylogenetic analysis. The scale bar represents the rate of nucleotide substitutions per site.

Partial sequences of the *16S rRNA* gene of *K*. *diezihuensis* ACCP0342 and *K*. *jiangxiensis* ACCP0444 were obtained. The phylogenetic tree based on *16S rRNA* gene sequences contained 84 nucleotide sequences with a total of 1,185 nt positions in the final dataset, further revealing the phylogenetic relationship between these two strains and their sister taxa (Figure 2). From the phylogenetic tree based on *16S rRNA* gene sequences, ACCP0342, ACCP0444, *K. muscicola*, *K. minuta*, “*Leptothermofonsia sichuanensis*,” *K*. *anagnostidisii*, *K*. *brockii*, and *K*. *atmophytica* were clustered into a group, and assigned to the *Kovacikia* genus, while other sister taxa *Chroakolemma*, *Pantanalinema,* and *Stenomitos* were significantly separated into individual lineages at the genus level (Figure 2) (Becerra-Absalón et al., 2018; Vaz et al., 2015; Miscoe et al., 2016; Shalygin et al., 2020). *Leptothermofonsia* Daroch, Tang and Shah 2022 was merge to *Kovacikia* Miscoe, Pietrasiak and Johansen 2016, while *Leptothermofonsia* is not considered a valid name, due to improper designation of the holotype (Miscoe et al., 2016; Tang et al., 2022; Kaštovský et al., 2023). Therefore, these sequences of “*L. sichuanensis*” are only used as background references for the *Kovacikia* genus.

Comparing the *16S rRNA* gene sequences of *K*. *diezihuensis* (1,481 bp) with *K*. *jiangxiensis* (1,482 bp) separately, the similarity was 97.2% (data not shown). Cyanobacteria with 98.7% or less *16S rRNA* gene sequence similarity may be considered different species (Yarza et al., 2014). Compared with *K. minuta*, the *16S rRNA* gene sequences of ACCP0342 and ACCP0444 shared 96.8 and 95.7% similarities, respectively (Table 2). The *16S rRNA* gene sequences (Table 2) of *K*. *diezihuensis* and *K*. *jiangxiensis* showed 94.9%–96.8% similarity with those of the other five *Kovacikia* species, indicating that they are distinct species within the same genus (Stackebrandt and Goebel, 1994; Sciuto et al., 2012; Kim et al., 2014; Komárek et al., 2014; Yarza et al., 2014; Sciuto and Moro, 2016; Eckert et al., 2015).

**Table 2:** Similarities of the *16S rRNA* gene sequences between the representative strains in this study (black bold part) and related sister taxa in the *Kovacikia* genus.

Sequence of strain	1	2	3	4	5	6	7	8	9	10
1. **ACCP0342**										
2. **ACCP0444**	98.2									
3. *Kovacikia muscicola* HA7619-LM3 clone 41A (KU161669.1)	96.4	95.3								
4. *K. muscicola* HA7619-LM3 clone 41B (KU161670.1)	96.2	95.0	99.5							
5. *Kovacikia minuta* CCNU0001 (OK110207.1)	96.8	95.7	97.5	97.3						
6. “*Leptothermofonsia sichuanensis*” PKUAC-SCTE412 (CP072600.1)	96.3	96.2	95.3	95.0	95.1					
7. *Kovacikia atmophytica* BACA0619 (OM732256.1)	96.7	96.1	97.1	96.9	96.9	96.0				
8. *Kovacikia anagnostidisii* YS86-RH1 (OR236708.1)	95.6	95.0	97.4	97.1	96.7	96.2	98.1			
9. *K. anagnostidisii* YS89-RH1 (OR236709.1)	95.5	94.9	97.3	97.0	96.6	96.1	98.1	99.9		
10. *Kovacikia brockii* YNP74-RH1 clone 1 (OR220391.1)	95.5	95.0	94.9	94.6	94.4	97.5	94.8	95.4	95.3	
11. *K. brockii* YS7-RH2 (OR236707.1)	96.1	95.6	95.4	95.1	95.0	98.1	95.4	96.0	95.9	99.3

### 3.2 *16S-23S* ITS analysis

The lengths of *16S–23S rRNA* ITS sequences of ACCP0342 and ACCP0444 strains were 493 bp and 488 bp, with two types of tRNA: tRNA^Ile^ (74 bp) and tRNA^Ala^ (73 bp), respectively. The *16S–23S rRNA* ITS sequences of the five representative strains, including the *K. muscicola* HA7619-LM3 clone 41A (KU161669.1), *K. minuta* CCNU0001 (OK110207.1), *K*. *anagnostidisii* YS86-RH1 (OR236708.1), *K*. *brockii* YNP74-RH1 clone 1 (OR220391.1), and *K*. *atmophytica* BACA0619 (OM732256.1), were complete with 617, 610, 550, 519, and 602 bp, respectively. The percent dissimilarity among *16S–23S rRNA* ITS region has been shown to be very effective at establishing cyanobacterial species (Boyer et al., 2001; Komárek et al., 2014; Osorio-Santos et al., 2014; Kaštovský et al., 2023). Compared with *K. minuta*, the 16S-23S ITS sequences of ACCP0342 and ACCP0444 shared 38.9 and 38.2% similarities, respectively (data not shown). The *16S–23S rRNA* ITS sequences of ACCP0342 and ACCP0444 strains showed 10.6% divergences with each other and 33.9 to 38.9% divergences with the other five *Kovacikia* species (data not shown), which matched the thresholds for different species (Becerra-Absalón et al., 2018; González-Reséndiz et al., 2018; Mai et al., 2018; Vázquez-Martínez et al., 2018; Mareš et al., 2019; Pietrasiak et al., 2019; Becerra-Absalón et al., 2020).

**Figure 3:**
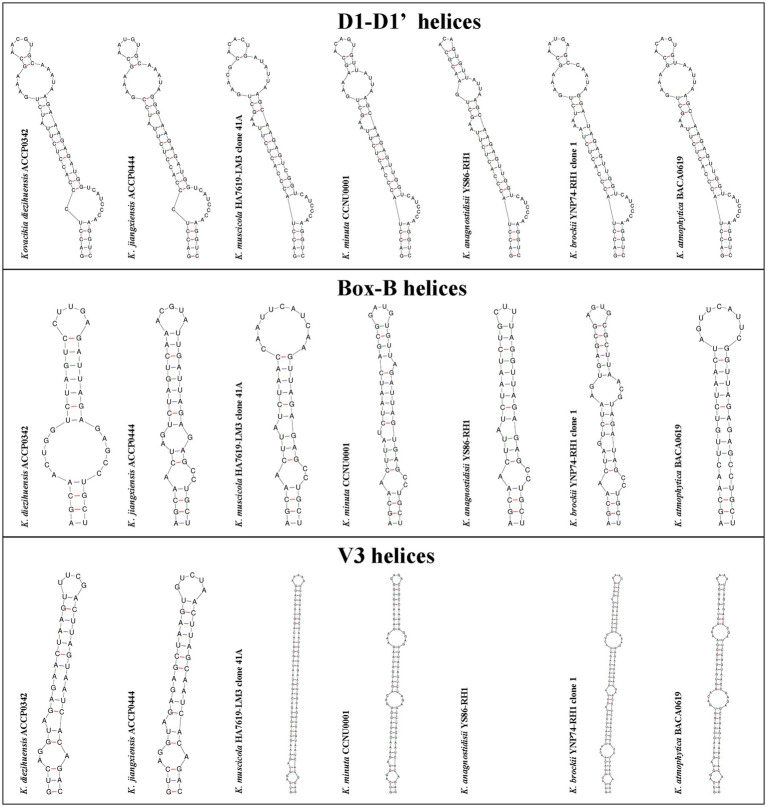
The representative regions of 16S-23S ITS secondary structure of D1-D1’, Box-B, and V3 helices about *Kovacikia diezihuensis* ACCP0342, *Kovacikia jiangxiensis* ACCP0444 strains, and other strains of *Kovacikia* species.

The secondary structures of representative regions of *16S–23S rRNA* ITS, namely, D1–D1’, Box-B, and V3 helices, are shown in Figure 3. ACCP0342 and ACCP0444 strains have similar lengths of D1–D1’ helix with 62 nucleotides. All strains had a basal stem (GACCU-AGGUC), with little difference in the middle and lower parts of the D1–D1’ helix. The main difference was concentrated at the top, where only three nucleotides differed between ACCP0342 and ACCP0444.

The length of the Box-B helix was 37–47 nucleotides. About the length of the Box-B helix, ACCP0342 was the shortest with 37 nucleotides, and ACCP0444 was 41 nucleotides, which was the same as *K. muscicola* HA7619-LM3 clone 41A (KU161669.1) and *K*. *atmophytica* BACA0619 (OM732256.1). The Box-B helix was partially identical at the bottom part (AGCA-UGCU), and the difference was greater in the upper part, especially at the top.

The V3 helix varied greatly overall in length (range 40–110 nucleotides) and secondary structure conformation. The V3 helix was absent in *K*. *anagnostidisii* YS86-RH1 (OR236708.1) (Figure 3). The V3 region of *K*. *anagnostidisii* YS86-RH1 (OR236708.1) was incomplete and contained 88 nucleotides, and its tail portion was missing, resulting in the failure to form a stable helical structure (Figure 3). The V3 helix of ACCP0342 was most similar to ACCP0444; both of them were short, 40 and 41 nucleotides in length, respectively, with only five base differences, and concentrated at the top. All V3 helices had the same basal stem structure (GUCAGGU-ACAGAC), and the V3 helices of ACCP0342 and ACCP0444 were generally very different from the other strains on the whole.

In summary, based on the differences in the *16S–23S rRNA* ITS sequences and their secondary structures, ACCP0342 and ACCP0444 exhibited uniqueness to be distinguished as different species.

### 3.3 Morphological description

**Figure 4:**
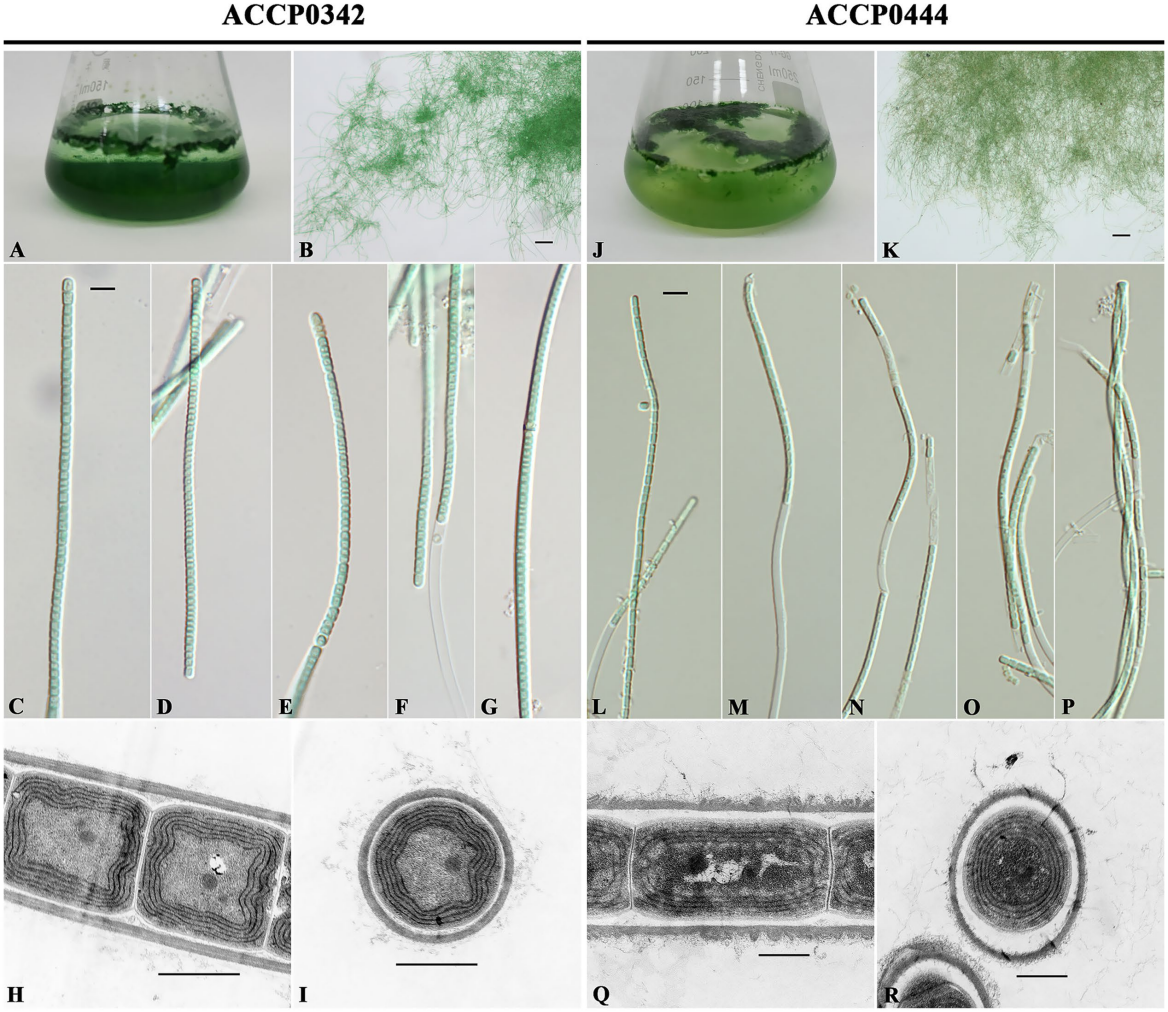
Morphological characteristics of *Kovacikia diezihuensis* ACCP0342 and *Kovacikia jiangxiensis* ACCP0444 strains on the white light (WL). **(A,J)** Morphological characteristics in a conical flask containing BG11 medium. **(B–G)** and **(K–P)** Differential interference contrast microscope images. **(B)** and **(K)** scale bar, 50 μm. **(C–G)** and **(L–P)** scale bar, 5 μm. **(H,I,Q,R)** Transmission electron micrographs. **(H,I)** Scale bar, 1 μm; **(Q,R)** Scale bar, 500 nm.

*Kovacikia diezihuensis* L.-Q. Shen & R. Li sp. nov. (Figures 4A–I).

*Diagnosis*: Its color was bright blue-green under WL, which was different from other *Kovacikia* species. Its *16S rRNA* gene sequence had a similarity of 97.2% with *K*. *jiangxiensis* ACCP0444, and a similarity of 95.5%–96.8% with five other species in the *Kovacikia* genus. It had unique secondary structures in the ITS region.

*Description*: In liquid culture, the filaments extended and entangled to form thin to slightly thick mats, most of which were attached to the bottom or small parts, and were clustered on the liquid culture surface and in contact with the bottle body. The color was bright blue-green under WL. The filaments were elongated, occasionally slightly curved, without false branches, heterocysts, and akinetes. The sheath was colorless, slightly thin, and occasionally visible. The trichomes were not attenuated to the ends, slightly constricted at the crosswalls, and occasionally had necridia. The cells were cylindrical, with rounded apical cells, without calyptras; sometimes small circular particles were observed in the center of the cells. The cells were isodiametrical or had a length slightly greater than or slightly less than their width, with 0.90–2.92 μm (mean 1.67 μm) length and 1.46–2.21 μm (average 1.86 μm) width. The cells had 4–6 layers of parietal thylakoid membrane. Reproduction occurred by hormogonia, trichome breakage via necridia, and subsequent disintegration, then releasing hormogonia.

*Etymology*: The specific epithet “diezihuensis” refers to the collection location of *K*. *diezihuensis* from Diezihu Avenue, Nanchang City, Jiangxi Province, China.

*Holotype*: ACCP-ZLJX20210342, a packet consisting of culture material of the ACCP0342 strain preserved at 4% formaldehyde in a 10 mL centrifuge tube and dried biomass of the same strain preserved in 2 mL frozen storage tubes, deposited at the ACCP, Jiangxi Key Laboratory of Flood and Drought Disaster Defense, JAWSE.

*Isotype*: WZUH-ZLJX20210342, a packet consisting of culture material of the ACCP0001 strain preserved at 4% formaldehyde in a 10 mL centrifuge tube and dried biomass of the same strain preserved in 2 mL frozen storage tubes, deposited at the College of Life and Environmental Sciences, Wenzhou University.

*Reference strain*: ACCP0342. Culture deposited at ACCP, Jiangxi Key Laboratory of Flood and Drought Disaster Defense, JAWSE.

*Habitat and type locality*: The strain was isolated from soil under the bamboo forest in the corner of a wall at Diezihu Avenue, Nanchang City, Jiangxi Province (28°41′12.13″N, 115°50′06.57″E).

*Other strain, habitat, and locality*: Strain ACCP0340 was isolated from moss-like soil in the corner of a wall at Diezihu Avenue, Nanchang City, Jiangxi Province (28°41′12.32″N, 115°50′05.56″E).

*Kovacikia jiangxiensis* L.-Q. Shen & R. Li sp. nov. (Figures 4J–R).

*Diagnosis*: Its color was gray-green to blue-green in color, and the cell length was usually longer than the width under the WL, which was different from other *Kovacikia* species. Its *16S rRNA* gene sequence had a similarity of 97.2% with *K*. *diezihuensis* ACCP0342, and a similarity of 94.9%–96.2% with five other species in the *Kovacikia* genus. It had unique secondary structures in the ITS region.

*Description*: In liquid culture, the filaments extended and entangled to form thin to slightly thick mats. Small parts were attached to the bottom, whereas large clusters of filaments floated on the liquid culture surface. The color was gray-green to blue-green under WL. The filaments were elongated, occasionally slightly curved, without false branches, heterocystes, and akinetes. The sheath was colorless, slightly thin, and occasionally visible. The trichomes were not attenuated to the ends, slightly constricted at the crosswalls, and occasionally had necridia. The cells were cylindrical, with rounded apical cells, without calyptras. The cells, usually with a length greater than their width, measure 1.11–2.92 μm (mean 1.92 μm) long and 0.96–1.49 μm (average 1.17 μm) wide. The cells had 3–5 layers of parietal thylakoid membrane. Reproduction occurred by hormogonia, trichome breakage via necridia and subsequent disintegration, then releasing hormogonia.

*Etymology*: The specific epithet “*jiangxiensis*” refers to the collection location of *K*. *jiangxiensis* from Jiangxi Normal University, Nanchang City, Jiangxi Province, China.

*Holotype*: ACCP-ZLJX20210444, a packet consisting of culture material of the ACCP0444 strain preserved at 4% formaldehyde in a 10 mL centrifuge tube and dried biomass of the same strain preserved in 2 mL frozen storage tubes, deposited at the ACCP, Jiangxi Key Laboratory of Flood and Drought Disaster Defense, JAWSE.

*Isotype*: WZUH-ZLJX20210444, a packet consisting of culture material of the ACCP0444 strain preserved at 4% formaldehyde in a 10 mL centrifuge tube and dried biomass of the same strain preserved in 2 mL frozen storage tubes, deposited at the College of Life and Environmental Sciences, Wenzhou University.

*Reference strain*: ACCP0444. Culture deposited at ACCP, Jiangxi Key Laboratory of Flood and Drought Disaster Defense, JAWSE.

*Habitat and type locality*: The strain was isolated from dried fluffy soil in the corner of Jiangxi Normal University, Nanchang City, Jiangxi Province, China (28°41′0.786″N, 116°1′50.467″E).

### 3.4 Pigment analysis

**Figure 5:**
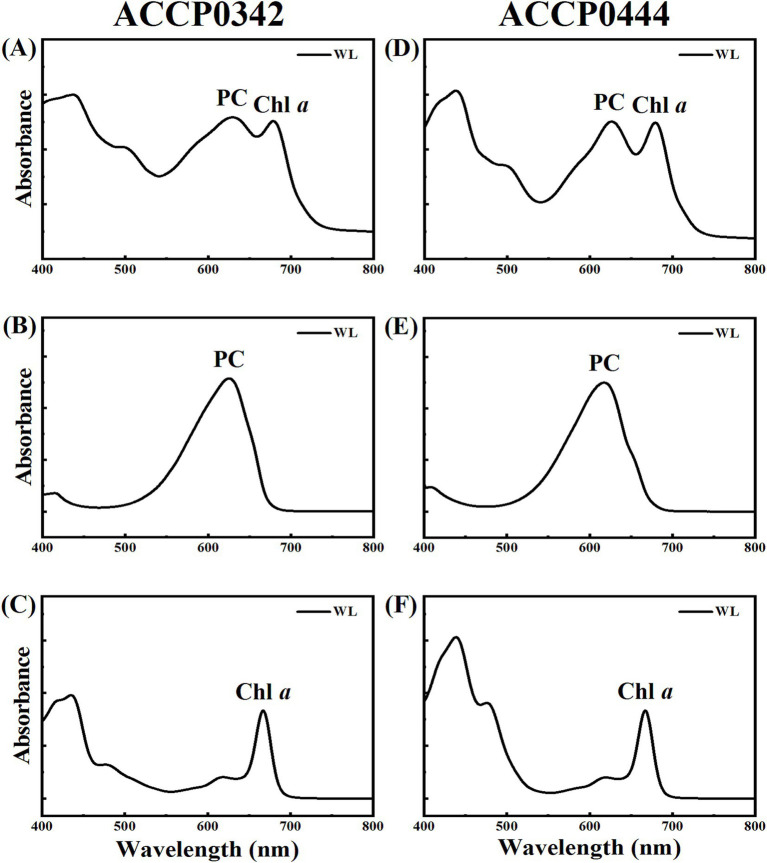
Pigment absorption spectra of *Kovacikia diezihuensis* ACCP0342 **(A–C)** and *Kovacikia jiangxiensis* ACCP0444 **(D–F)** strains. The solid line shows white light (WL). **(A,D)** Cell absorption spectrum was homogenized according to the absorption peak of Chl *a* (680 nm); **(B,E)** Absorption spectra of water-soluble pigments were homogenized with the absorption peak of PC (618 nm); **(C,F)** Fat-soluble absorption spectrum, homogenized with the absorption peak of Chl *a* in methanol (667 nm).

The absorption spectra for ACCP0342 and ACCP0444 strains under WL are shown in Figure 5. The pigments mainly included Chl *a*, phycocyanin (PC), and carotenoids by the cell absorption spectrum (Figures 5A,D). The water-soluble pigment analysis (Figures 5B,E) showed that ACCP0342 and ACCP0444 strains produced a large amount of PC (absorption peak around 618 nm under WL), but did not produce phycoerythrin (PE). Based on the absorption spectrum of lipid-soluble pigments (Figures 5C,F), ACCP0342 and ACCP0444 strains produced Chl *a* (absorption peak around 667 nm in methanol under WL). The two strains could not produce red-shifted complexes and Chl *f* induced by FRL and could not grow under FRL (Figure 5).

## 4 Discussion

According to previous reports, the species of *Kovacikia* were widely distributed, being found in Jiangxi Province, Hubei Province and Sichuan Province in China; across the Eurasian continent to the Azores Archipelago (Portugal) in the Atlantic Ocean, and across Yellowstone National Park (USA) in North America, as well as the Hawaiian Islands in the Pacific Ocean (Miscoe et al., 2016; Shen et al., 2022; Kaštovský et al., 2023), which across through the whole Northern Hemisphere, and are all in the mid-latitude zone (Table 1). This also illustrates the geographical universality of species in the *Kovacikia* genus. Despite their geographically distant distribution, their ability to reproduce in a diverse range of habitats, including freshwater, atmophytic environment attachment beneath plants, cave walls, soil surfaces, and hot springs, reflects their strong adaptability to different conditions. These species were filaments that formed mat-like groups, microbial mats, mosses, or crusts, which may help them adapt to environmental changes. In addition, the *Kovacikia* species were widely distributed and had diverse habitats, most likely a ubiquitous class of cyanobacteria present in the environment.

*Kovacikia* was first identified as a new genus by a polyphasic approach, which is mostly defined by its unique phylogenetic position and the secondary structure of the ITS region (Miscoe et al., 2016). Molecular genetics methods are commonly used in a polyphasic approach and are crucial in the modern cyanobacterial classification. In this study, the phylogenetic analysis showed that they were clustered in the *Kovacikia* genus with the other five *Kovacikia* species. Comparing the *16S rRNA* gene sequences (Table 2), *K*. *diezihuensis*, *K*. *jiangxiensis*, and the other five *Kovacikia* species shared 94.9%–97.2% similarity, which belongs to the appropriate threshold for different species in the same genus (Stackebrandt and Goebel, 1994; Sciuto et al., 2012; Kim et al., 2014; Komárek et al., 2014; Yarza et al., 2014; Eckert et al., 2015; Sciuto and Moro, 2016). Compared to other *Kovacikia* species, the secondary structures of the ITS regions of *K. diezihuensis* and *K. jiangxiensis* exhibit significant differences, demonstrating their uniqueness. All these molecular genetic data support the separation of *K. diezihuensis* and *K. jiangxiensis* from the other five *Kovacikia* species.

Actually, *Kovacikia* is considered to be similar to *Leptolyngbya* (Anagnostidis and Komárek, 1988) in morphology, making it difficult to distinguish (Miscoe et al., 2016). In this study, the most obvious morphological difference between *K. diezihuensis* and other *Kovacikia* species is that its color is bright blue-green. In contrast, other species are either purple-brown or gray-green to blue-green under WL. Secondly, its cell length is slightly smaller than the average width, which is similar to that of *K*. *anagnostidisii* (Kaštovský et al., 2023). *K. Jiangxiensis* is the most similar to *K*. *atmophytica* (Luz et al., 2023) in morphological characteristics, but there is still a slight difference in that its cell width is slightly smaller. *K. Jiangxiensis* is different from other *Kovacikia* species in color or cell length and width (Supplementary Table S2). Therefore, the two new species, *K*. *diezihuensis* and *K*. *jiangxiensis*, were similar to the other five *Kovacikia* species in morphology. At the same time, they have fine distinctions in color and length-to-width ratio in the cell.

Among the five officially reported and effectively named *Kovacikia* species, only *K. minuta* was reported to produce Chl *f*. “*Leptothermofonsia*” was merged with *Kovacikia*, while “*Leptothermofonsia*” is not considered a valid name, due to improper designation of the holotype (Kaštovský et al., 2023). However, “*L. sichuanensis*” E412, a strain of *Kovacikia*, can also grow, and the color was green under FRL. Combined with genome data, it is speculated that the strain also has the ability to produce Chl *f*. In terms of habitat, *K. minuta* was isolated from the colony underneath macrophytes in a shaded pond, “*L. sichuanensis*” E412 was isolated from the colony in the pond of Lotus Lake Hot Spring, while *K*. *diezihuensis* and *K*. *jiangxiensis* in this study were isolated from moss-like soils in the corner. These habitats are common habitats for Chl *f*-producing cyanobacteria (Antonaru et al., 2020), suggesting that there may be niche overlap between Chl *f*-producing cyanobacteria and non-Chl *f*-producing cyanobacteria. The results of Ohkubo and Miyashita suggest that the deeper layer of the microbial mat was a habitat for Chl *f*-producing cyanobacteria, and Chl *f* enabled them to survive in a habitat with little PAR (Ohkubo and Miyashita, 2017). Therefore, in microbial mats or colonies, the non-Chl *f*-producing cyanobacteria may be closer to WL, while the Chl *f*-producing cyanobacteria are closer to FRL.

In terms of molecular genetics, the phylogenetic tree showed that they clustered in the *Kovacikia* genus (Figure 2). The non-Chl *f-*producing *K*. *diezihuensis* and *K*. *jiangxiensis* in this study diverged at the species level from *K. minuta* and “*L. sichuanensis*” E412, and had 95.0%–96.8% similarity with *16S rRNA* gene sequences (Table 2). Furthermore, the secondary structures of the ITS regions and ITS sequences exhibit significant differences, demonstrating their uniqueness (Figure 3). All these show that both Chl *f*-producing and non-Chl *f*-producing species at the same genus in *Kovacikia.* This provides significant impetus for further analysis of the evolutionary divergence of Chl *f-*producing cyanobacteria and their adaptability to the different environments.

Compared to *K*. *diezihuensis*, in morphology, *K*. *jiangxiensis* is more similar to *K. minuta* and “*L. sichuanensis*” E412, both of which have slender cells. In *Kovacikia*, both *K. minuta* and “*L. sichuanensis*” E412, which produce Chl *f* and contain PE, are brown or purplish-brown under WL and grass-green or green under FRL(Shen et al., 2022; Tang et al., 2022). However, *K*. *diezihuensis* and *K*. *jiangxiensis*, which do not produce Chl *f* and do not contain PE, are bright blue-green or blue-green to gray-green under WL. It is speculated that *K*. *muscicola* containing PE may also have the ability to produce Chl *f*. Among the reported *Leptolyngbya*-like cyanobacteria producing Chl *f*, it was found that all other cyanobacteria contain phycoerythrin except *Leptodesmis undulata*. Therefore, Chl *f* may be more easily found in *Leptolyngbya*-like cyanobacteria containing phycoerythrin.

## 5 Conclusion

In summary, three *Leptolyngbya*-like cyanobacteria were phylogenetically identified as *K. diezihuensis* sp. nov. (reference strain, ACCP0342) and *K. jiangxiensis* sp. nov. (reference strain, ACCP0444) (Leptolyngbyaceae, Leptolyngbyales) using a polyphasic approach combining ecological, molecular, and morphological data, respectively. These two new species of *Kovacikia* did not produce phycoerythrin under WL and Chl *f* under the induction of FRL. This is the first report of both Chl *f*-producing and non-Chl *f*-producing species within the same genus in the Leptolyngbyales, shedding light on the diversity and the evolutionary divergence of Chl *f*-producing cyanobacteria.

In addition, their habitats are common habitats for Chl *f*-producing cyanobacteria, suggesting that there may be niche overlap between Chl *f*-producing cyanobacteria and non-Chl *f*-producing cyanobacteria. Among the reported *Leptolyngbya*-like cyanobacteria producing Chl *f*, it was found that all other cyanobacteria contain phycoerythrin except *Leptodesmis undulata*. Therefore, it is assumed that Chl *f* may be more easily found in *Leptolyngbya*-like cyanobacteria containing phycoerythrin. These discoveries can help elucidate the evolution of FaRLiP in cyanobacteria and provide a new perspective for the ecological application of Chl *f* cyanobacteria with its FaRLiP gene cluster in the future.

## Data Availability

The datasets presented in this study can be found in online repositories. The names of the repository/repositories and accession number(s) can be found at: https://www.ncbi.nlm.nih.gov/genbank/, PQ881585; PQ895252; PQ881586.
