# Correction: Two new non-chlorophyll *f*-producing species in the *Kovacikia* genus (Leptolyngbyaceae, Leptolyngbyales) from the Poyang Lake Basin, China

**DOI:** 10.3389/fmicb.2025.1711378

**Published:** 2025-10-14

**Authors:** Frontiers Production Office

**Affiliations:** Frontiers Media SA, Lausanne, Switzerland

**Keywords:** cyanobacteria, *Leptolyngbya*-like, *Kovacikia*, new species, polyphasic approach, chlorophyll *f*

There was a mistake in Figure 2 as published. “*Kovacikia diezihuensis ACCP0342*” should be “*Kovacikia diezihuensis* ACCP0342” and “*Kovacikia jiangxiensis ACCP0444”* should be “*Kovacikia jiangxiensis* ACCP0444”.

The corrected figure appears below.

**Figure 2:**
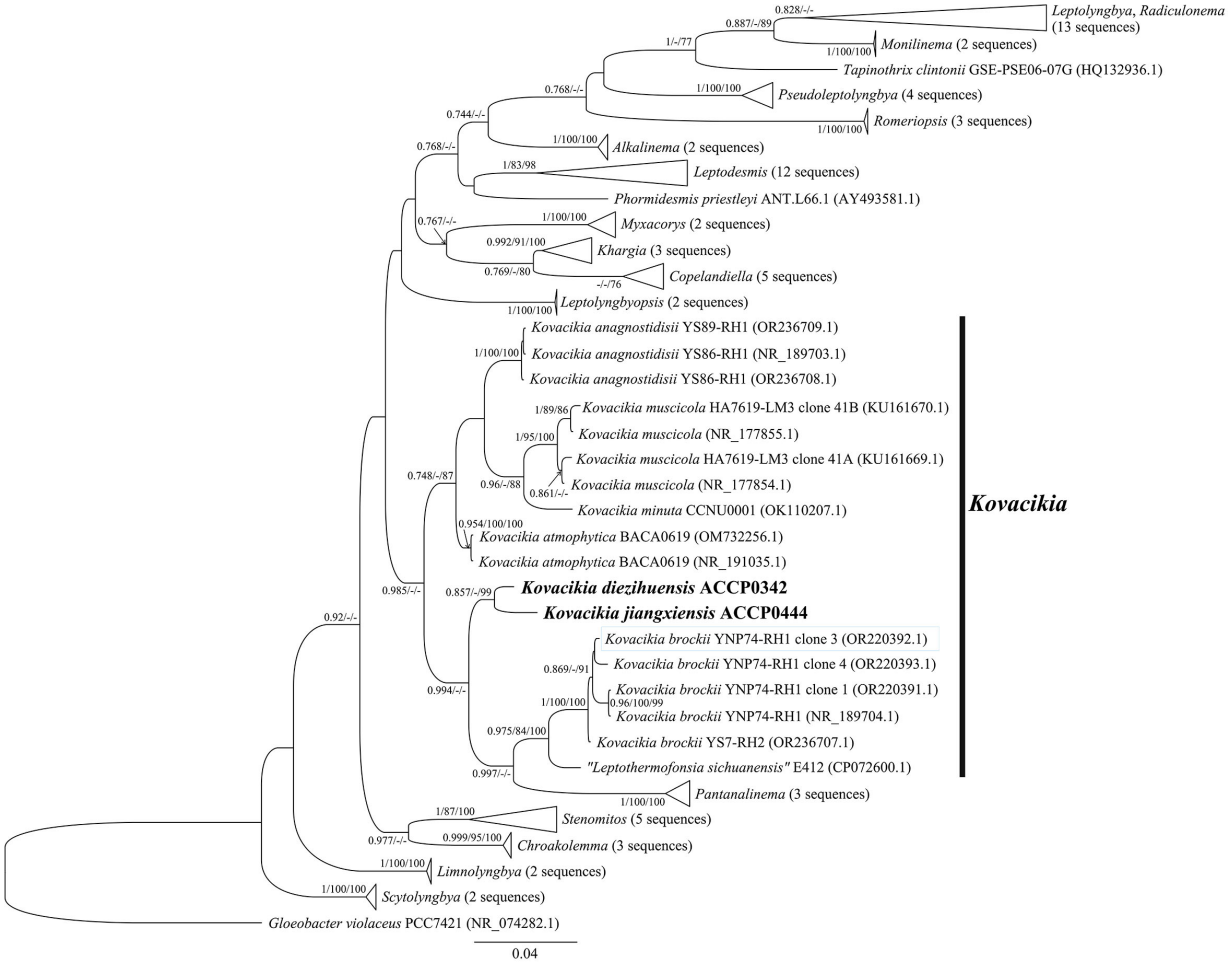
Bayesian phylogenetic tree based on the *16S rRNA* gene sequences showing the relationships of *Kovacikia diezihuensis* ACCP0342 and *Kovacikia jiangxiensis* ACCP0444 (bold part) with their similar taxa. The nodes marked by black dots were genus horizontal, and the black square nodes were horizontal nodes of the Leplyngbyaceae family. Bootstrap values greater than 70% were given in front of the corresponding nodes for BI/ML/NJ phylogenetic analysis. The scale bar represents the rate of nucleotide substitutions per site.

There was a mistake in Figure 3 as published. The first two strains in each of the three sections were incorrectly labeled.

The corrected figure appears below.

**Figure 3:**
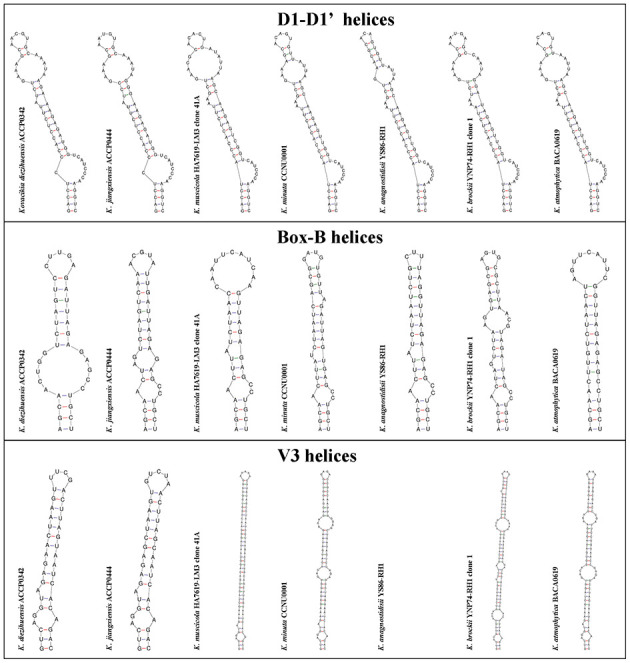
The representative regions of 16S-23S ITS secondary structure of D1–D1′, Box-B, and V3 helices about *Kovacikia diezihuensis* ACCP0342, *Kovacikia jiangxiensis* ACCP0444 strains, and other strains of *Kovacikia* species.

The original version of this article has been updated.

