# Supplementary material for: Two new non-chlorophyll f-producing species in the Kovacikia genus (Leptolyngbyaceae, Leptolyngbyales) from the Poyang Lake Basin, China
Source: Front Microbiol. 2025 Sep 22;16:1578689. doi: 10.3389/fmicb.2025.1578689 (PMC12497795; doi:10.3389/fmicb.2025.1578689)
Supplement: Supplementary file 1 [file Presentation_1.pdf]

Supplementary Material

1 Supplementary Tables

Table S1. Sequence information of strains.

Species names	Strain numbers	Accession numbers	Sequence lengths
Kovacikia diezihum	ACCP0342	PQ881585	1969 bp
Kovacikia diezihum	ACCP0340	PQ895252	1970 bp
Kovacikia jiangxia	ACCP0444	PQ881586	1975 bp

Table S2. Summary of morphological characteristics in *Kovacikia* genus. “No= Not available.”

Species	***Kovacikia diezihum* (ACCP0342)**	***K. jiangxia* (ACCP0444)**	*K. muscicola* (HA7619-LM3)	*K. minuta* CCNU0001	*K. anagnostidisii* (YS86-RH1)	*K. brockii* (YNP74-RH1)	*K. atmophytica* (BACA0619)
Colony	In liquid culture, filaments extend and cross-wound to form thin to slightly thick mats, most of them attached to the bottom or small parts is clustered on the liquid culture surface and the contact with the bottle body.	In liquid culture, filaments extend and cross-wound to form thin to slightly thick mats. Small parts are attached to the bottom, while large clusters of filaments float on the liquid culture surface.	Colony a spreading mat, penetrating the agar, dark purplish brown.	Formed mats in liquid culture, often attached to the bottom or floating on the surface.	Colony a flat mat, with entangled filaments.	Colony a flat mat, with entangled filaments.	Colony a flat mat, with entangled filaments.
Color	Bright blue-green under white light	Grey-green to blue-green under white light.	Grayish green to purplish brown in color.	Purplish-brown under white light but became grass green under far-red light.	Green to yellow-green, grey-green or blue-green.	Grey-green to blue green, pale blue-green to green.	In young cultures blue green, and greenish to brownish when old.
Filament	Elongated, occasionally slightly curvature, without false branch, heterocyst and akinete.	Elongated, occasionally slightly curvature, without false branch, heterocyst and akinete.	Lacking pseudobranching, sometimes with a widened sheath containing one to two coiled trichomes, up to 3.2 µm wide.	Isopolar, solitary, lacked heterocysts, or were coiled into clusters and mats.	Filaments slightly coiled, sometimes spirally coiled, without false branching, 1.5–2.5 µm wide.	Filaments straight, coiled, sometimes spiral, without false branching, 1.3–2.7 µm wide	Filaments normally straight or slightly waived, without false branching, 1.4–2.0 µm wide (mean = 1.68 µm).
Sheath	Colorless, slightly thin, and occasionally visible.	Colorless, slightly thin, and occasionally visible.	Thin, colorless.	Thin and colorless.	Thin, colorless, attached to the trichome, scarcely visible.	Thin, colorless, attached to the trichome.	Thin, colourless, attached to the trichome.

Trichomes	Not attenuated to the ends, slight constricted at the crosswalls, occasional necridia.	Not attenuated to the ends, slight constricted at the crosswalls, occasional necridia.	Trichomes untapered, slightly to distinctly constricted at the crosswalls.	Individual trichomes were fine, cylindrical, usually did not attenuate at their ends, and had slight constriction at crosswalls.	Trichomes not attenuated, distinctly constricted at cross walls, lacking necridia, immotile, granulated.	Trichomes not narrowed toward the end, constricted or slightly constricted at cross walls, often disintegrating into hormogonia in the absence of necridia, lacking motility, often with granulation in the middle of cell.	Trichomes untapered, slightly constricted at cross walls, without necridia, without motility. Hormogonia few celled, with motility.
Cell	Cells cylindrical, with rounded apical cells, without calyptras; sometimes small circular particles can be seen in the center of the cells. Cells isodiametrical or length slightly greater than width or slightly less than width, with 0.90-2.92 µm (mean 1.67 µm) long and 1.46-2.21 µm (average 1.86 µm) wide.	Cells cylindrical, with rounded apical cells, without calyptras. Cells usually length greater than width, with 1.11-2.92 µm (mean 1.92 µm) long and 0.96-1.49 µm (average 1.17 µm) wide.	Cells homogenous, mostly a little longer than wide, 1.5–1.7 µm wide, 1.0–2.0 µm long	Had rounded apical cells, cells were 0.93–2.17 µm long and 1.14–1.31 µm wide.	Apical cells rounded. Cells mostly shorter than wide or almost isodiametric, (1.3) 1.6–2.0 (2.4) µm wide and 1–2.1 (2.5) µm long.	Apical cells rounded. Cells more or less isodiametric in younger filaments, or shorter in old trichomes, (0.9) 1.1–2.5 µm wide, 0.8–2.2 µm long, older trichomes are distinctly wider that young trichomes.	Cells mostly longer than wide or almost isodiametric, sometimes varying within the same trichome, with parietal thylakoids, 1.0–1.6 µm wide (mean = 1.30 µm) to 1.1–2.9 µm long (mean = 1.94 µm), with a length:width ratio of 0.8–2.1 (mean = 1.49). End cells rounded.
Thylakoid	4-6 layers parietal thylakoid membranes.	3-5 layers parietal thylakoid membranes.	Parietal thylakoids.	Four to six parietal thylakoid membranes.	No	No	No
Reference	This study	This study	Miscoe et al. 2016	Shen et al. 2022	Kaštovský et al. 2023	Kaštovský et al. 2023	Luz et al. 2023
